# Macrophages in ulcerative colitis: A perspective from bibliometric and visual analysis

**DOI:** 10.1016/j.heliyon.2023.e20195

**Published:** 2023-09-17

**Authors:** Lijiang Ji, Qiong Zhou, Jinke Huang, Dongxue Lu

**Affiliations:** a Department of Anorectal Surgery, Changshu Hospital Affiliated to Nanjing University of Chinese Medicine, Changshu, 215500, Jiangsu, China; b Graduate School, Beijing University of Chinese Medicine, Beijing, 100105, China; c Department of Integrative Oncology, China-Japan Friendship Hospital, Beijing, 100029, China; d Xiyuan Hospital of China Academy of Chinese Medical Sciences, Beijing, 100091, China; e Department of Nutrition, Acupuncture and Moxibustion and Massage College & Health Preservation and Rehabilitation College, Nanjing University of Chinese Medicine, Nanjing, 210023, Jiangsu, China

**Keywords:** Global trends, Macrophages, Ulcerative colitis, Bibliometrics

## Abstract

**Objectives:**

Despite the many reported studies on macrophages in ulcerative colitis (UC), the overall research trends in this field are unclear. This study evaluates the research trends and hotspots regarding macrophages in UC using bibliometric analysis.

**Methods:**

A systematic search was conducted in the Web of Science database to identify publications related to macrophages in UC from 2012 to 2021. R package ‘bibliometrix’, VOSviewers, CiteSpace and Microsoft Excel were utilised for the bibliometric analysis.

**Results:**

1074 articles published between 2012 and 2021 were analysed. The number of publications on macrophages in UC showed a consistently increasing trend, with USA and China as the leading contributors to this field. Notably, Georgia State University and Nanjing University contributed significantly to this field. Among the authors, Wang Y had the highest productivity, while Wu X received the most citations. The journal *Gut* was identified as the most authoritative journal in this field. Co-citation analysis revealed that the exploration of the mechanisms of macrophages in UC through in vivo and in vitro experiments was the primary focus of research. Moreover, the emerging research hotspots included keywords such as ‘macrophage polarization’, ‘gut microbiota’ and ‘NLRP3 inflammasome’.

**Conclusions:**

Research on macrophages in UC holds significant value and practical implications. Additionally, China demonstrated prolific output in this field, while the USA had the most influential contributions. Currently, research hotspots are centred around the modulation of gut microbiota to regulate macrophage polarization and macrophage pyroptosis as potential strategies for mitigating UC.

## 1 Introduction

Ulcerative colitis (UC) is a rapidly increasing immune-mediated inflammatory disease worldwide [1]. Although the aetiology of UC remains unknown, it is speculated that environmental triggers, genetic factors, intestinal bacteria and immune responses contribute to the pathogenesis of UC [2]. UC is pathologically characterised by abnormal mucosal immunoreactivity and persistent inflammatory infiltrates. Mounting evidence suggests that both innate and adaptive immune abnormalities contribute to abnormal inflammatory responses in the gut [3]. Innate immunity elicits nonspecific responses, while sustained inflammation triggers adaptive immunity, potentially leading to chronic inflammation [4]. During the development and progression of UC, macrophages, which exhibit significant diversity and plasticity, can polarise into classical M1 or alternative M2 types with different functional phenotypes [5,6]. Macrophage-driven immune responses have been demonstrated to play a crucial role in the pathogenesis of UC in humans and also mouse models of colitis [7,8]. Macrophages are, therefore, critical for regulating immune homeostasis in the intestinal mucosa [9]. A quantitative analysis of the current understanding of macrophages in UC is vital for identifying key areas and prospects.

Bibliometrics is a quantitative analysis method that explores trends and hotspots in a research field through the objective analysis of published papers [10]. By examining the knowledge base and research frontiers using data from published articles, bibliometrics provides valuable evidence to guide experimental strategies and funding decisions [11]. Currently, it has become widely used and accepted in the scientific community [12]. However, a bibliometric study focusing on macrophages in UC is currently lacking. Therefore, to fill this gap and extract important, valid and meaningful information from large databases to assess the current state and hotspots in this field, we conducted this bibliometric study.

## 2 Materials and methods

### 2.1 Data source and search strategy

A search was conducted in the Web of Science database on 26 August 2022. The search strategy used the following terms: TS = (Ulcerative colitis OR idiopathic proctocolitis OR ulcer colonitis OR colitis gravis) AND TS = (macrophage OR macrophages). The inclusion criteria were as follows: (a) literature published between 2012 and 2021; (b) articles as the type of literature; and (c) literature published in English. Duplicate publications were excluded. A manual check of the included literature was independently performed by two authors.

### 2.2 Data acquisition

Following a systematic search of the Web of Science database, full records and cited references of the publications were exported and converted to a. txt format. Additionally, the original data from the Web of Science, including affiliations, publication year, the number of papers and citations, countries/regions, journals and H-index, were recorded. Finally, the obtained data were analysed.

### 2.3 Bibliometric analysis

R software (4.0.2), VOSviewer (1.6.18), CiteSpace (6.1.R2) and Microsoft Excel (2019) were utilised for generating data tables and visual knowledge graphs. The analysis of the number of publications, citations, countries/regions, affiliations, authors, journals, and topics trend was performed using the R-package (http://www.bibliometrix.org). To acquire more comprehensive results, bibliometric maps based on co-citation and co-occurrence were constructed using VOSviewer. CiteSpace was applied to identify keywords with strong citation bursts. Additionally, the polynomial-fitting curve was generated using Microsoft Excel.

## 3 Results

### 3.1 Publication characteristics

**Fig. 1:**
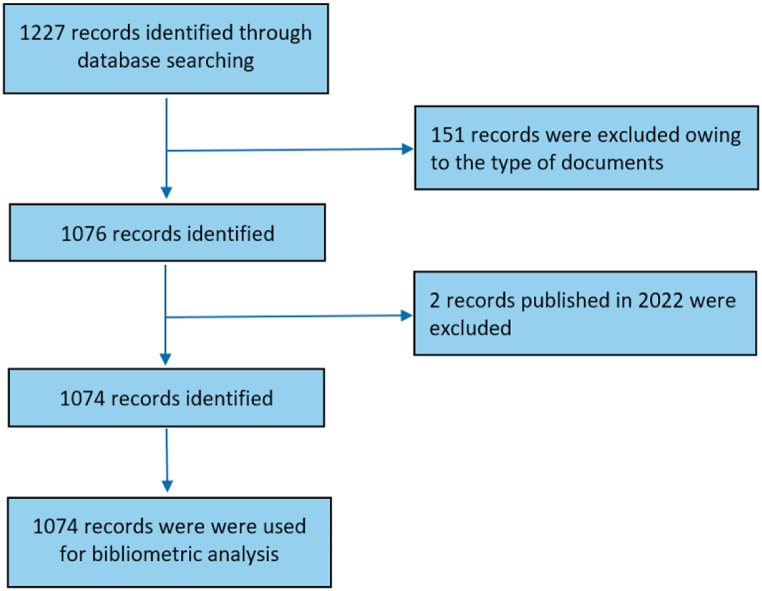
Flow diagram of the literature selection process.

**Fig. 2:**
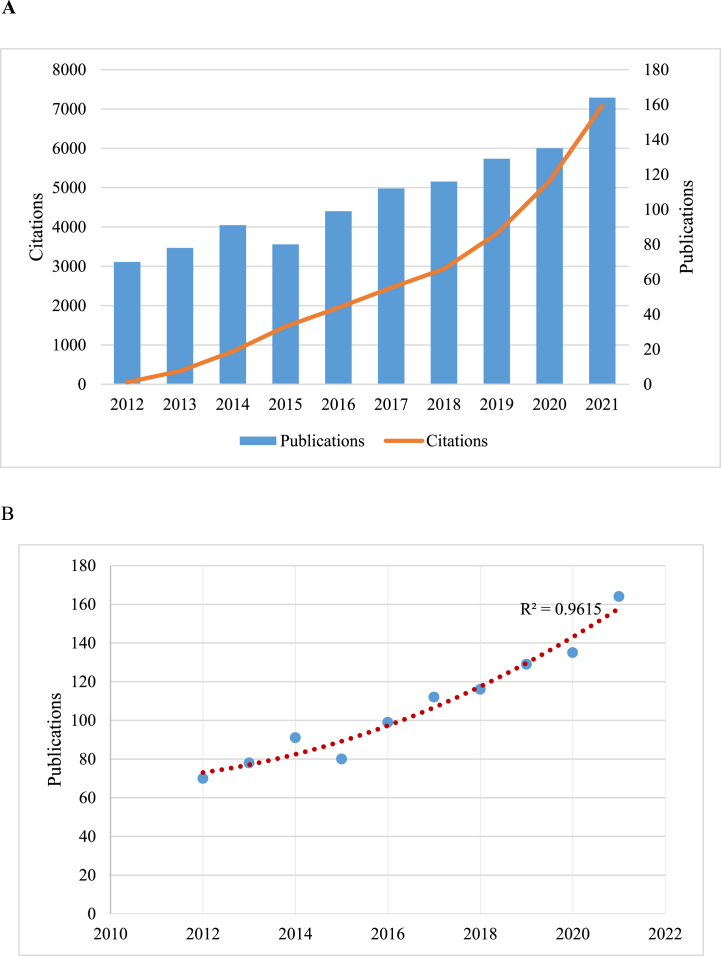
(A) The number of publications and annual citations over the past 10 years. (B) Curve fitting of the e total annual growth trend of publications (R^2^ = 0.9615).

1227 publications were identified during the preliminary search. On screening the titles and abstracts of the identified publications, 1074 publications met the inclusion criteria and were selected for further data extraction. Fig. 1 shows the flow chart of literature identification. The annual publication trend related to macrophages in UC is presented in Fig. 2A. Despite some fluctuations, the number of annual publications showed a steady upward trend, increasing from 70 in 2012 to 164 in 2021. Fig. 2B displays the polynomial-fitting curve, which shows an association between the number of annual publications and the publication year (correlation coefficient (R^2^) = 0.9574. Similarly, the number of citations for these publications also showed a steady increase, rising from 70 in 2012–7087 in 2021. These findings suggest that research on macrophages in UC is a popular and rapidly developing field.

### 3.2 Analysis of countries/regions

**Fig. 3:**
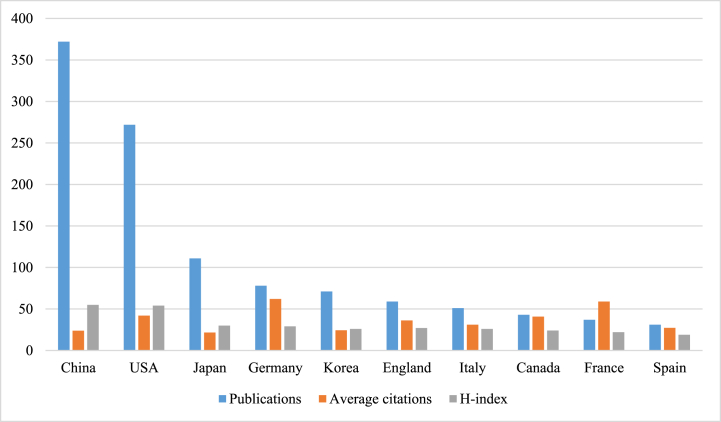
The co-citation frequency and H-index for the top 10 productive countries/regions.

**Fig. 4:**
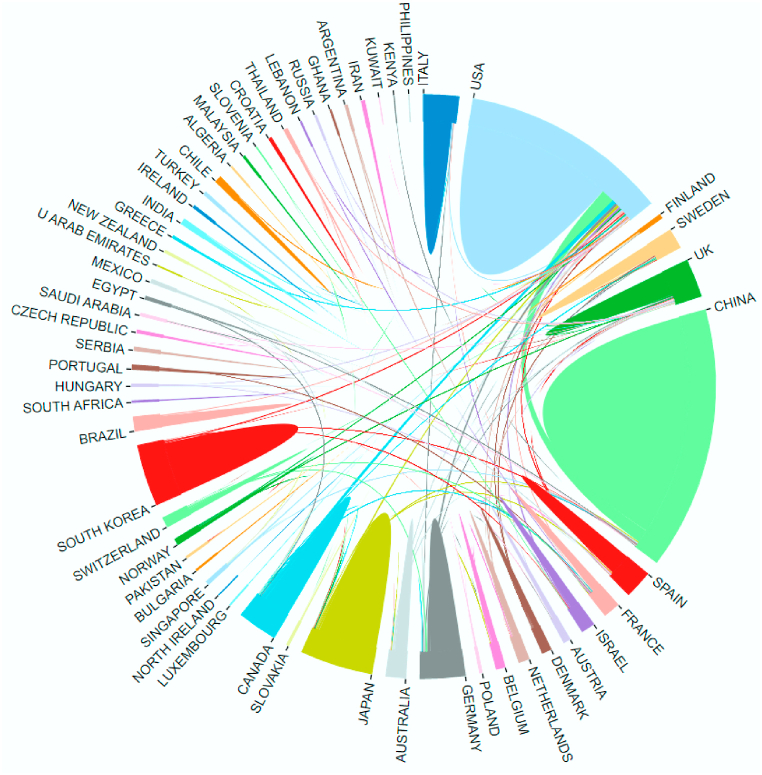
The international collaboration between countries/regions. Note that the different colours of arcs represent different countries/regions, and the larger the arc area, the wider the international cooperation of the country/region. Line thickness between countries/regions reflects the intensity of the closeness.

The 1074 publications included were contributed by 65 different countries or regions. Table 1 and Fig. 3 illustrate the productivity of the countries, with China having the highest number of publications (272, 34.64%), the USA having the highest total citations (n = 11406) and Germany having the highest average citations (n = 61.99). Notably, international cooperation was prevalent in China, the USA, Japan and Korea (Fig. 4).

**Table 1:** The top 10 productive countries/regions.

Ranking	Country/Region	Publications	% of (1,074)	Total citations	Average citations	H-index
1	China	372	34.64	8861	23.82	55
2	USA	272	25.33	11406	41.93	54
3	Japan	111	10.34	2406	21.68	30
4	Germany	78	7.26	4835	61.99	29
5	Korea	71	6.61	1725	24.30	26
6	England	59	5.49	2135	36.19	27
7	Italy	51	4.75	1584	31.06	26
8	Canada	43	4.00	1753	40.77	24
9	France	37	3.45	2180	58.92	22
10	Spain	31	2.89	846	27.29	19

### 3.3 Analysis of affiliations

**Fig. 5:**
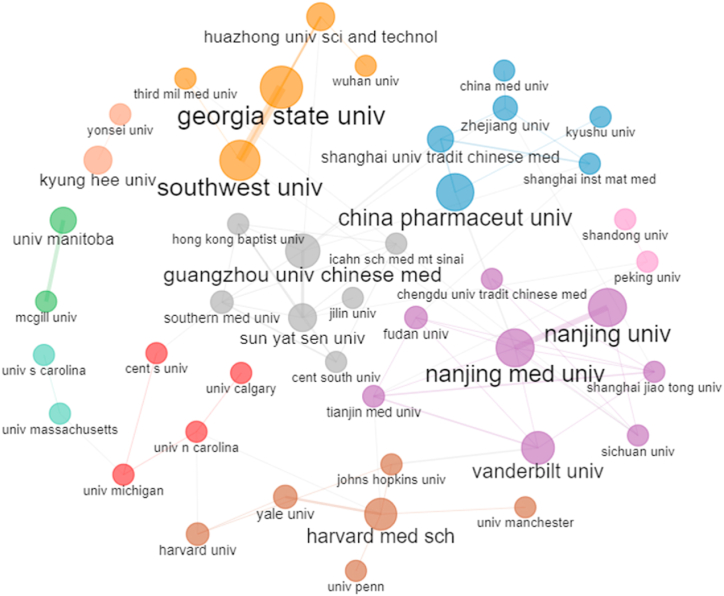
Collaboration between affiliations. Note that each circle represents an affiliation, and the larger the circle, the wider the cooperative relationship. Affiliations with frequent cooperative relationships are clustered into plates of the same colour. Line thickness between affiliations reflects the intensity of the closeness.

The 1074 publications were affiliated with 1569 different institutions. As shown in Table 2, Georgia State University (n = 20) and Nanjing University (n = 20) were the most productive affiliations. Vanderbilt University affiliations produced the highest total citations (n = 850) and average citations (n = 53.13). Additionally, a high level of cooperation was found among Georgia State University, Southwest University, China Pharmaceutical University, Nanjing Medical University and Nanjing University (Fig. 5).

**Table 2:** The top 10 productive affiliations.

Ranking	Affiliation	Country	Publications	Total citations	Average citations
1	Georgia State University	USA	20	834	41.70
2	Nanjing University	China	20	769	38.45
3	China Pharmaceutical University	China	19	588	30.95
4	Southwest University	China	16	741	46.31
5	Vanderbilt University	USA	16	850	53.13
6	Nanjing Medical University	China	16	774	48.38
7	KyungHee University	Korea	16	460	28.75
8	Harvard Medical School	USA	15	300	20.00
9	Guangzhou University of Chinese Medicine	China	15	258	17.20
10	University of Manitoba	Canada	13	271	20.85

### 3.4 Analysis of authors

**Fig. 6:**
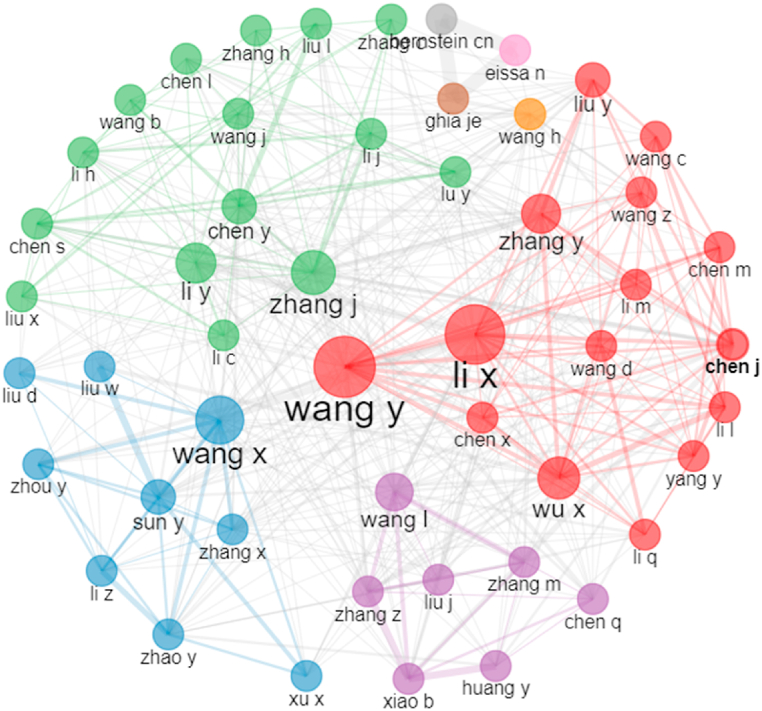
Collaboration between authors. Note that each circle represents an author, and the larger the circle, the wider the cooperative relationship. Authors with frequent cooperative relationships are clustered into plates of the same colour. Line thickness between authors reflects the intensity of the closeness.

The 1074 publications involved 7656 authors. As shown in Table 3, Wang Y was the most productive author (n = 37), while Wu X had the highest total citations (n = 1242), average citations (n = 54.00) and H-index (19). Notably, the top 10 authors were from China, and a high level of cooperation was reported by Wang Y, Li X and Wang X (Fig. 6).

**Table 3:** The top 10 productive authors.

Ranking	Author	Country	Publications	Total citations	Average citations	H-index
1	Wang Y	China	37	849	22.95	18
2	Li X	China	34	894	26.29	12
3	Zhang J	China	26	508	19.54	12
4	Li Y	China	25	545	21.80	14
5	Wu X	China	23	1242	54.00	19
6	Wang X	China	23	482	20.96	14
7	Liu Y	China	21	445	21.19	10
8	Wang L	China	19	860	45.26	14
9	Zhang Y	China	19	698	36.74	11
10	Zhang J	China	26	508	19.54	12

### 3.5 Analysis of journals

**Fig. 7:**
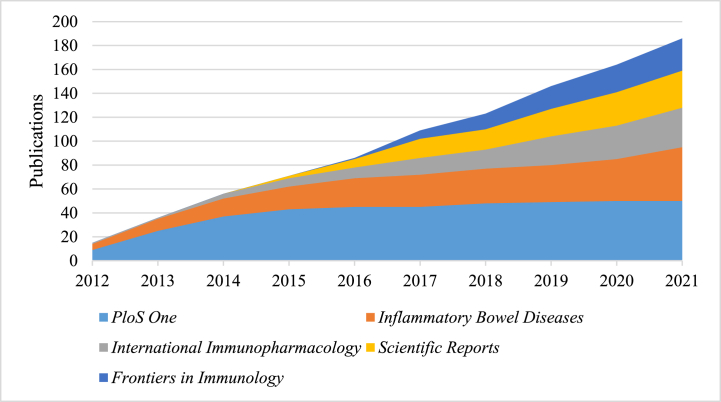
The number of publications of the top five most active journals over the past 10 years.

The 1074 publications were distributed across 383 journals. As shown in Table 4, *PloS One* had the highest number of publications (n = 50) and total citations (n = 1764). Additionally, *Gut* had the highest average citations (n = 87.88) and impact factor (31.793). Furthermore, the number of annual publications in the top five most active journals showed a consistent upward trend (Fig. 7).

**Table 4:** The top 10 most active journals.

Ranking	Journal	Publications	Total citations	Average citations	2021 IF
1	*PloS One*	50	1764	35.28	3.752
2	*Inflammatory Bowel Diseases*	42	1169	27.83	7.290
3	*International Immunopharmacology*	32	1136	35.50	5.714
4	*Scientific Reports*	30	800	26.67	4.996
5	*Frontiers in Immunology*	27	597	22.11	8.786
6	*Journal of Crohns & Colitis*	26	751	28.88	10.020
7	*World Journal of Gastroenterology*	23	1261	54.83	5.374
8	*Journal of Immunology*	18	595	33.06	5.426
9	*Gut*	16	1406	87.88	31.793
10	*Mucosal Immunology*	14	509	36.36	8.701

### 3.6 Analysis of Co-cited references

**Fig. 8:**
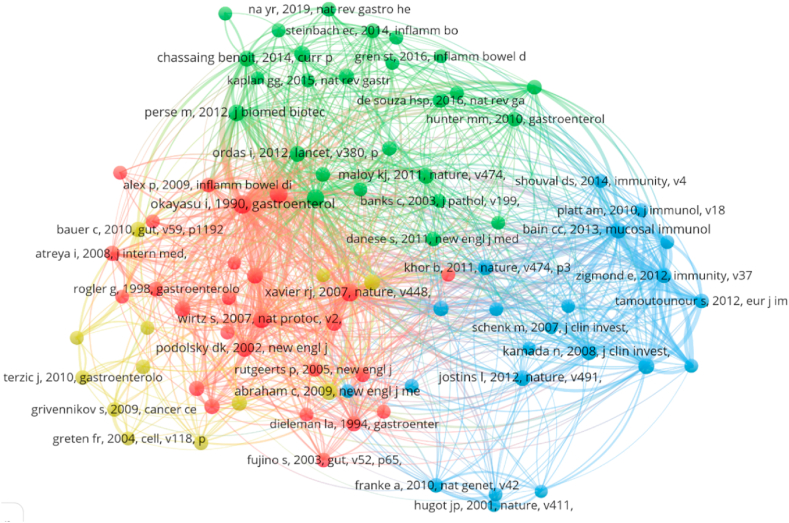
Network visualization map of co-citation references. Note that the lines between the circles represent the co-citation relationship. The thickness and number of connections between the nodes indicate the strength of links between references.

As shown in Fig. 8, cluster 1 (red) comprised 26 references focusing on clinical characteristics and animal models of UC. Cluster 2 (green) comprised 26 references that explored the regulatory effects of diverse macrophage populations on UC. Cluster 3 (blue) comprised 22 references that centred on the mechanisms and pathways of the inflammatory stage of UC. Finally, cluster 4 (yellow) comprised 12 references that examined the role of the inflammasome in UC.

### 3.7 Analysis of hotspots and trends in research

**Fig. 9:**
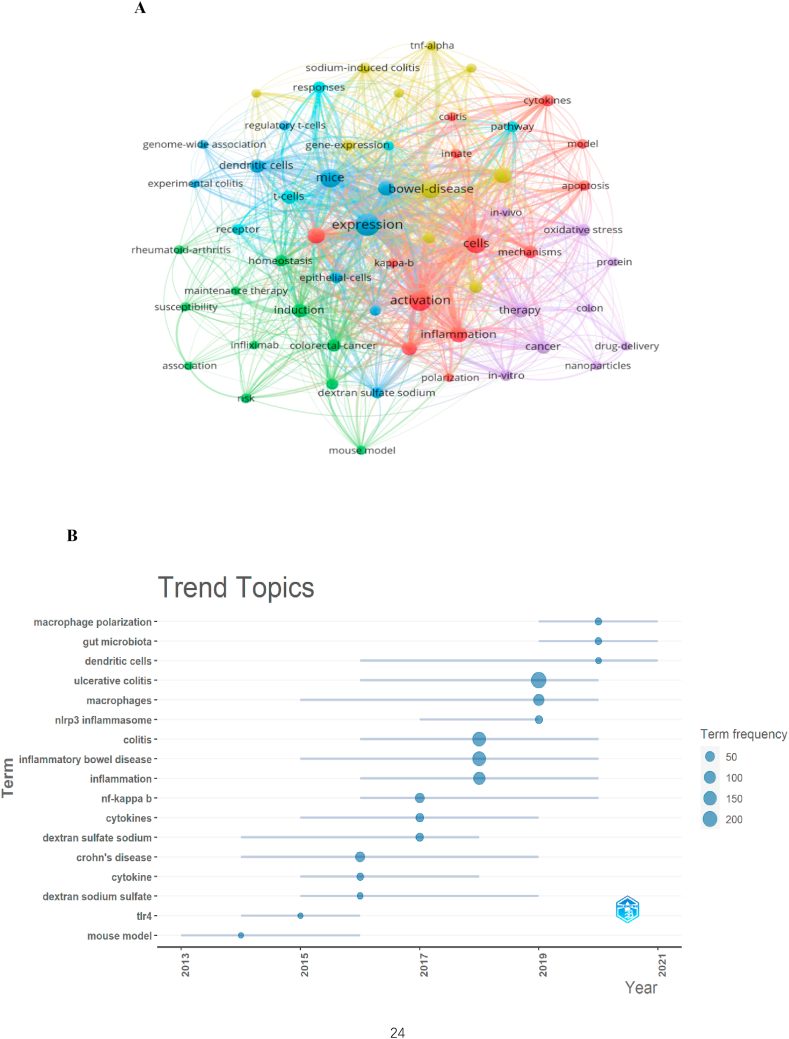

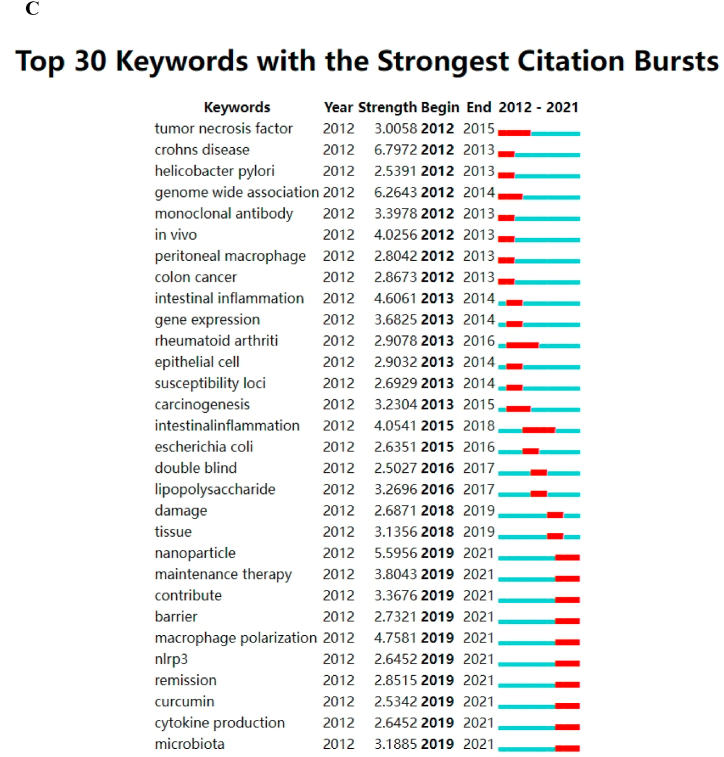
(A) Network map of keywords on macrophages in UC. Note that the node size reflects the co-occurrence frequencies and the link indicates the co-occurrence relationship. The thickness of the link is proportional to the number of times two keywords co-occur. (B) The trend of topics over time. Note that the length of the line suggests the length of the period in which the keyword appears; the circle represents the frequency of the keyword, and the larger the circle, the higher the frequency of the keyword. (C) Visualization map of top 30 keywords with the strongest citation bursts. The blue bars indicate that the keywords have been published and the red bars indicate citation burstness.

As shown in Fig. 9A, cluster 1 (red) and cluster 4 (yellow) primarily focused on in vitro studies of macrophages associated with UC. Meanwhile, cluster 2 (green) centred on in vivo studies of macrophages associated with UC. Cluster 3 (blue) and cluster 6 (cyan) focused on the treatment of UC and UC-related cancers. Cluster 5 (purple) revolved around UC caused by other factors. Excluding commonly used keywords such as macrophages, ulcerative colitis and inflammatory bowel disease, the top frequently occurring keywords were ‘expression’, ‘NF- κB’, ‘activation’, ‘mice’ and ‘cells’, suggesting that research on macrophages in UC predominantly focused on basic studies. Fig. 9B highlights the recent major topics in this field, namely ‘macrophage polarization’, ‘gut microbiota’, ‘dendritic cells’ and ‘NLRP3 inflammasome’. Additionally, CiteSpace identified the top 30 keywords with the strongest citation bursts (Fig. 9C), revealing emerging keywords, such as ‘nanoparticle’, ‘maintenance therapy contribute’, ‘barrier’, ‘macrophage polarization’, ‘nlrp3’, ‘remission’, ‘curcumin’ and ‘cytokine productionmicrobiota’. Thus, these findings indicate that ‘macrophage polarization’, ‘NLRP3 inflammasome’ and ‘gut microbiota’ are potential future research hotspots.

## 4 Discussion

The potential clinical application value of macrophages in UC garnered increasing attention with the increasing understanding of their role. Macrophages have emerged as a significant area of research in UC and a better understanding of their involvement in UC is essential for future research. Therefore, this study presents a bibliometric analysis of research trends and hotspots regarding macrophages in UC based on the Web of Science database.

### 4.1 Summary of main findings

Firstly, the number of publications and citations on macrophages in UC has been increasing over the last decade. Polynomial fitting curves indicate a steady upward trend in the annual number of publications, suggesting that macrophages have captured researchers' interest in the field of UC. Secondly, China and the US emerged as the most influential countries in this field. In the number of publications, China ranks first, and five of the top 10 productive affiliations coming from China. Furthermore, the top 10 productive authors originate from China. The USA ranks second in the number of publications; however, it has the highest number of citations compared to China, indicating a deeper exploration of macrophage research in UC. Thirdly, international collaboration plays an important role in the rapid development of this field. For example, USA and China exhibited a diverse range of cooperation, and the most influential affiliations in the US also had partnerships with various affiliations in China. Additionally, the most influential authors are majorly from China, and a close partnership was reported between them. Fourthly, high-quality authoritative journals are an important medium for facilitating the exchange of research results and promoting the in-depth development of research. Eight of the top 10 most productive journals reported a high IF (>5), indicating their interest in macrophages in UC and their important role in this field. Importantly, researchers interested in this area should focus more on these journals as they could provide a theoretical basis for future research. In particular, *Gut* should be recommended as an authoritative journal of interest to investigators in this field. Lastly, the understanding of the pathological features of UC and basic research based on animal models serve as the primary foundations in this research field. The co-cited references highlight four topics: clinical features and animal models of UC, the pathological mechanism of UC, the regulatory effect of different macrophage populations on UC and the role of inflammasomes in UC. Furthermore, inflammation caused by the hyperactivation of macrophages in UC has always been a research hotspot. The trend topic analysis and keywords with strong citation bursts revealed that ‘macrophage polarization’, ‘gut microbiota’ and ‘NLRP3 inflammasome’ have frequently focused on in the past 2 years and are therefore likely to represent the current research hotspots and trends of macrophages in UC.

### 4.2 Research hotspots and emerging topics

Macrophage polarization, gut microbiota and NLRP3 inflammasome were identified as the current research hotspots and trends in macrophages in UC. Macrophages are important regulators of intestinal immune homeostasis. The different polarization states of intestinal macrophages promote inflammation or anti-inflammation. Under normal conditions, the polarization of M1/M2 macrophages protects the intestine from inflammatory damage. However, under the influence of relevant genetic and environmental factors, dysregulation of the polarization of M1/M2 macrophages shifts the function from physiological immune protection of the intestine to pathological inflammatory damage. Therefore, deeper insights into the mechanisms of macrophage polarization in UC and the development of therapeutic strategies targeting macrophage polarization are gaining attention [13,14]. The integrity of the intestinal mucosal barrier depends on its interaction with the gut microbiota. Dysregulated bacterial communities have been strongly associated with the pathogenesis of UC, and the modulation of the gut microbiota has been demonstrated to have significant benefits for UC [15]. The involvement of intestinal flora in the pathophysiological processes of UC is complex. Recent evidence report that UC is mainly associated with the dysregulation of dynamic crosstalk between microbial metabolites and macrophages [16]. For instance, butyrate was found to have an essential role in host microbiota-macrophage communication [16]. Therefore, targeting the microbial metabolite butyrate could alleviate UC by modulating macrophage polarization [16,17]. Macrophage pyroptosis is another mechanism in the pathological progression of UC. Activation of the NLRP3 inflammasome in macrophages induces proinflammatory cytokines IL-1β and IL-18, which are important pathogenic factors in the development of UC [18]. Interestingly, a crosstalk between the gut microbiota and macrophage pyroptosis has been demonstrated. Moreover, the activation of the NLRP3 inflammasome in macrophages driven by the dysregulation of the gut microbiota was identified as an important mechanism in the development of various diseases [[19], [20], [21]], including intestinal inflammation [22]. Furthermore, the regulation of gut microbiota and inhibition of the NLRP3 inflammasome has been shown to be effective in UC [23,24]. Therefore, based on these findings, we hypothesise that gut microbiota, macrophage polarization and NLRP3 inflammasome are current research hotspots in this field, and further exploration of their crosstalk and potential clinical benefits will be future research trends.

### 4.3 Implications for research and practice

Mechanistically, the pathological mechanisms of the involvement of macrophage polarization, NLRP3 inflammasome, or gut microbiota in UC remain unclear. However, macroscopically, three broad pathways that regulate macrophage polarization, namely extrinsic factors (such as microbial products and cytokines), tissue microenvironment and cell survival and epigenetic pathways that shorten or prolong macrophage development and viability, have been reported [25]. However, from a microscopic perspective, the fine regulatory mechanisms of macrophage polarization are still lacking, including the upstream pathways regulating this process, the subsequent reflections triggered by downstream effector targets and the interactive regulatory networks formed with other molecules [26]. Similarly, the factors triggering the development of pyroptosis and its control mechanisms require further exploration. With the advancements in high-throughput sequencing technology, 16sRNA sequencing and macro-genome sequencing technologies have been widely applied to the deeper study of intestinal flora, revealing the Phylum, Class, Order, Family and Genus of the flora and providing more evidence to explore the participating roles of the flora in UC. The effect of the identified differential gut microbes and their metabolites or the interaction of the microbes with their surrounding community are potential topics for future research. Overall, based on the detailed exploration of the role of macrophage polarization, NLRP3 inflammasome and gut microbiota in the regulatory mechanisms of UC, it is crucial for current researchers in this field to further investigate the pathological mechanisms of UC around the intestinal microbe-macrophage polarization axis and intestinal microbe-macrophage pyroptosis axis.

These findings suggest that gut microbes play a key role in UC, with macrophage polarization and macrophage pyroptosis acting as downstream effects of gut microbial dysregulation. Therefore, the targeted modulation of gut microbial dysregulation could be a potentially promising therapeutic strategy to alleviate UC. From a clinical practice perspective, faecal microbiota transplantation (FMT), which aims to regulate intestinal flora, has shown positive effects in alleviating UC. Furthermore, evidence-based medical research indicates that FMT can lead to clinical and endoscopic improvement in the short-term treatment of active UC [27]. The faecal quality of donors, the volume of infusions, and the method of administration impact the effect of FMT, therefore, FMT protocols should be standardised to ensure clinical applicability. Additionally, the administration method, total dose, frequency and donor selection of FMT should also be investigated using larger-scale experiments.

### 4.4 Limitations

To our knowledge, this is the first bibliometric analysis that focuses on the research trends of macrophages in UC. However, some limitations must be acknowledged. In addition to the Web of Science database, other databases such as PubMed, Embase and Scopus also provide scientific literature. Moreover, publications such as conference abstracts and reviews were excluded, which could have potentially led to the omission of certain hotspots.

## 5 Conclusion

This study highlights the significant research value and potential application of investigation macrophages in UC. China emerged as a major contributor in terms of research output, while the USA exhibited many prominent breakthroughs in this field. Furthermore, the topic of alleviating UC by regulating gut microbiota as a means to modulate macrophage polarization or macrophage pyroptosis is becoming a research hotspot.

## Author contribution statement

All authors listed have significantly contributed to the development and the writing of this article.

## Data availability statement

Data will be made available on request.

## Additional information

No additional information is available for this paper.

## Declaration of competing interest

The authors declare that they have no known competing financial interests or personal relationships that could have appeared to influence the work reported in this paper.
